# Supplementary figures and images for: Extracellular Vesicles of Patients on Peritoneal Dialysis Inhibit the TGF-β- and PDGF-B-Mediated Fibrotic Processes
Source: Cells. 2024 Mar 29;13(7):605. doi: 10.3390/cells13070605 (PMC11011990; doi:10.3390/cells13070605)

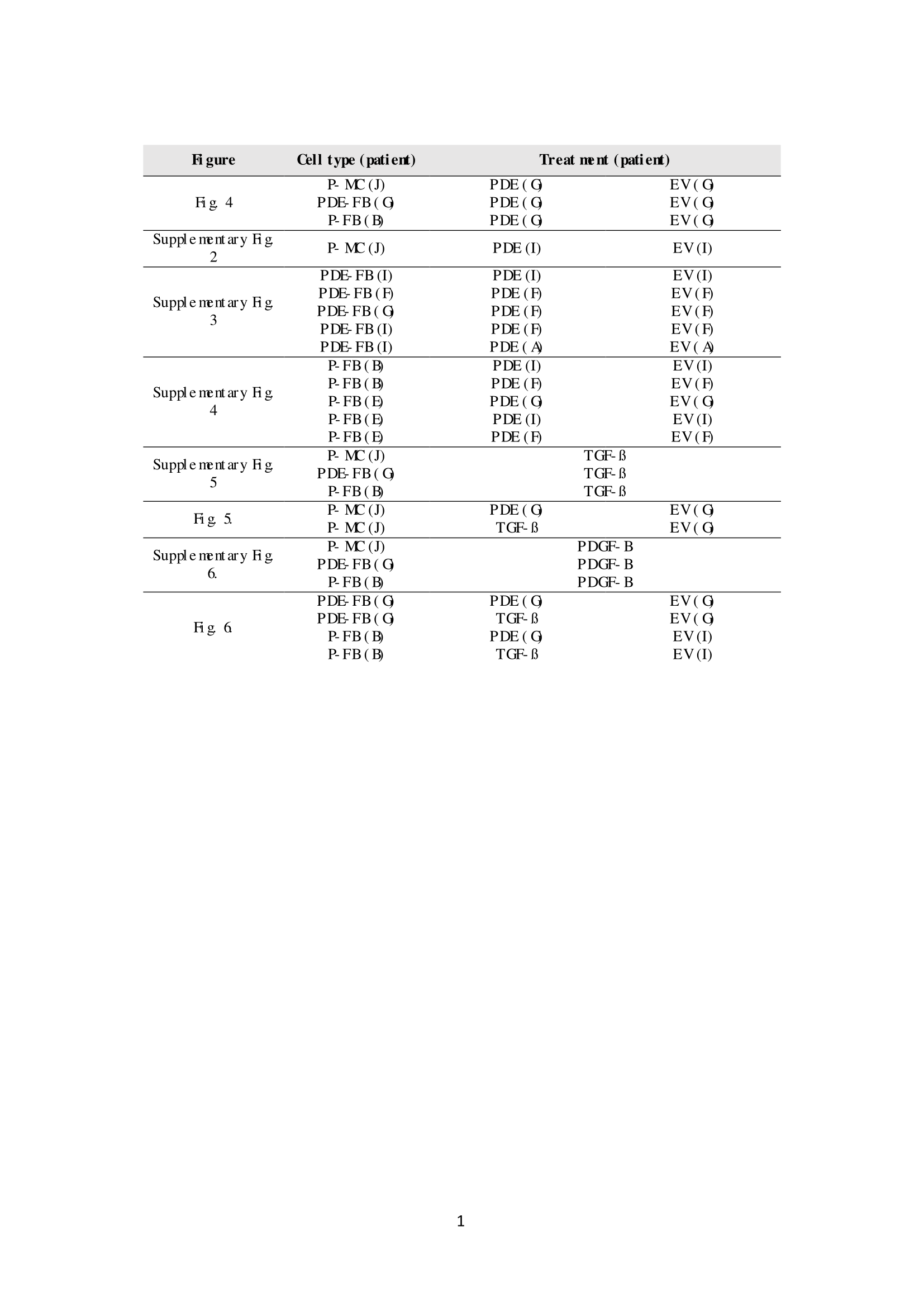

Supplement: Supplementary file 1 [file cells-13-00605-s001.zip › Figure S1.tiff]

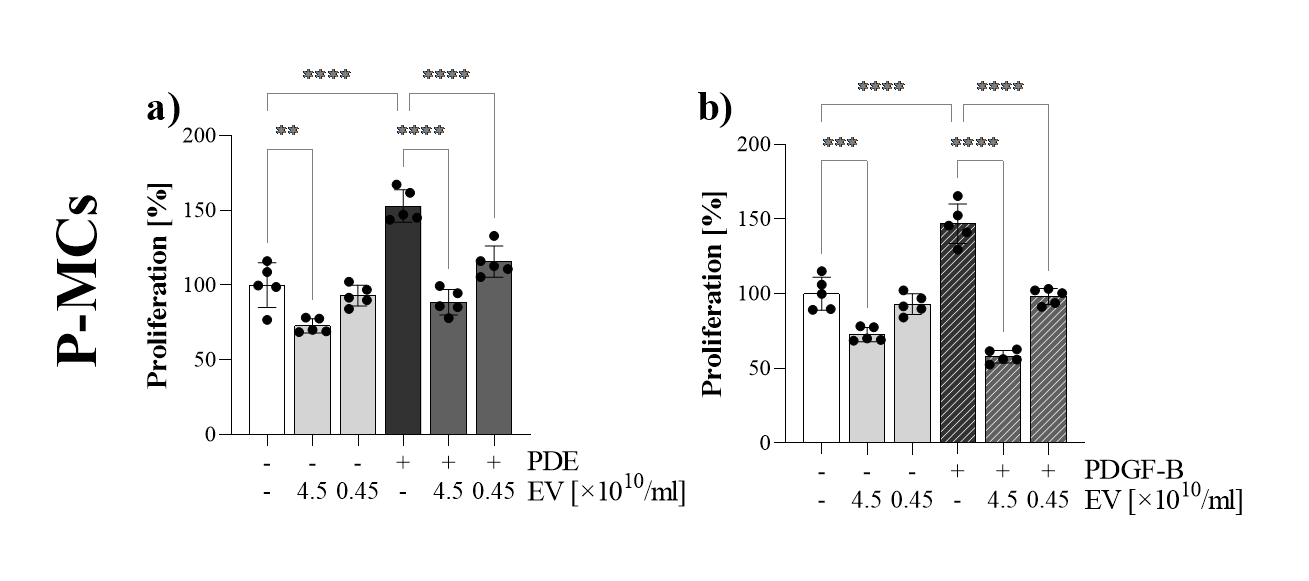

Supplement: Supplementary file 1 [file cells-13-00605-s001.zip › Supl 2.tif]

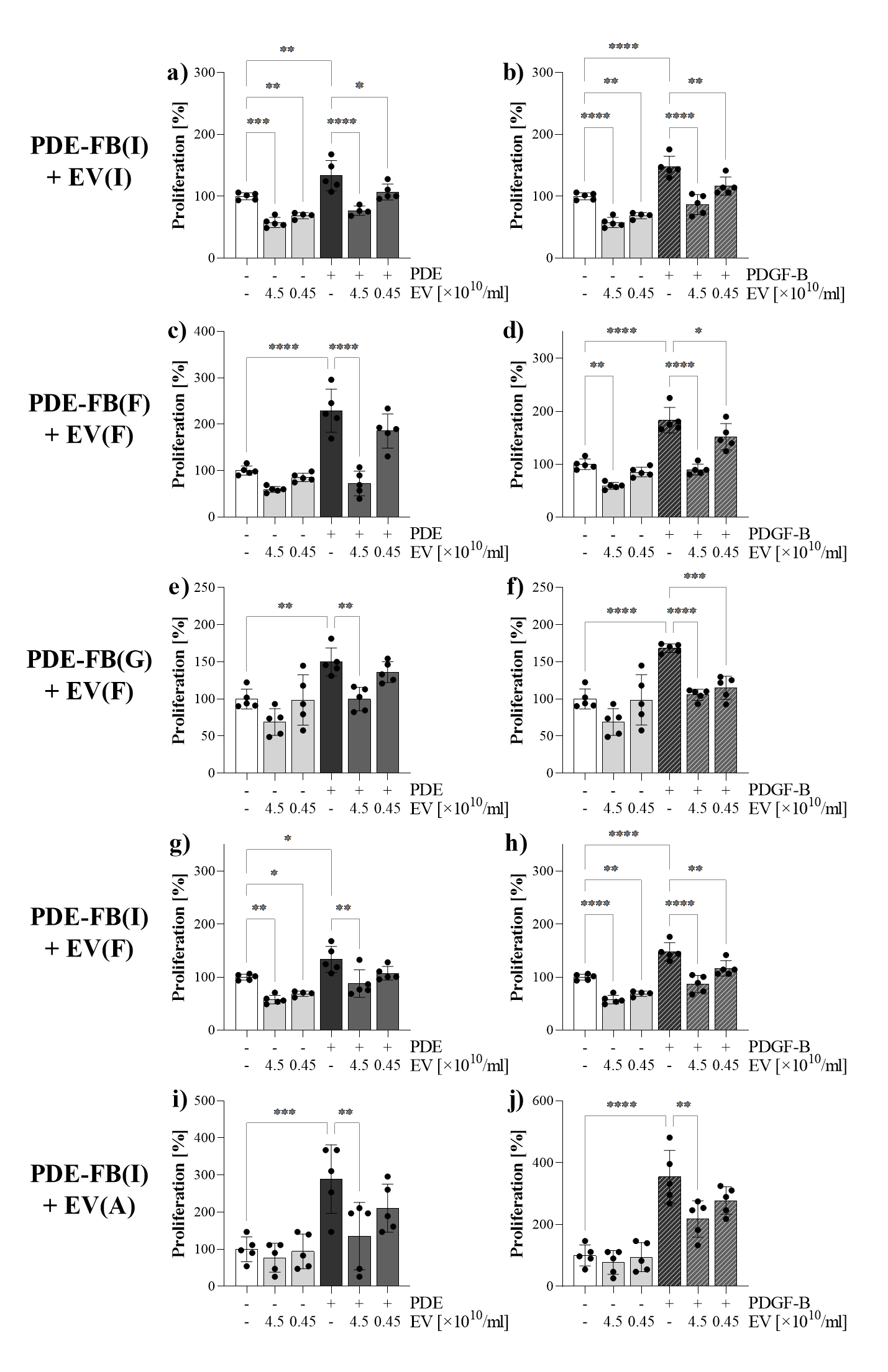

Supplement: Supplementary file 1 [file cells-13-00605-s001.zip › Supl 3.tif]

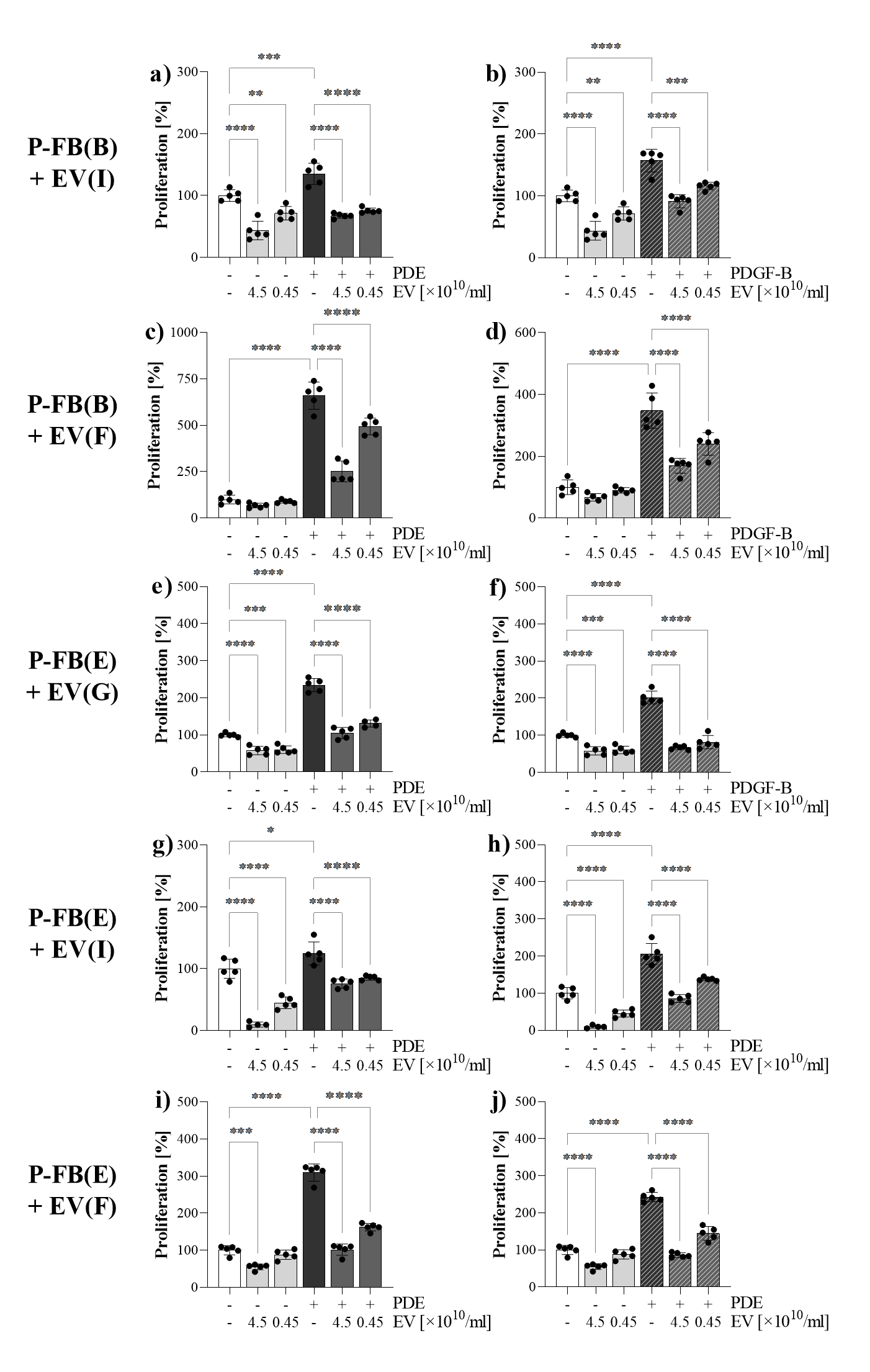

Supplement: Supplementary file 1 [file cells-13-00605-s001.zip › Supl 4.tif]

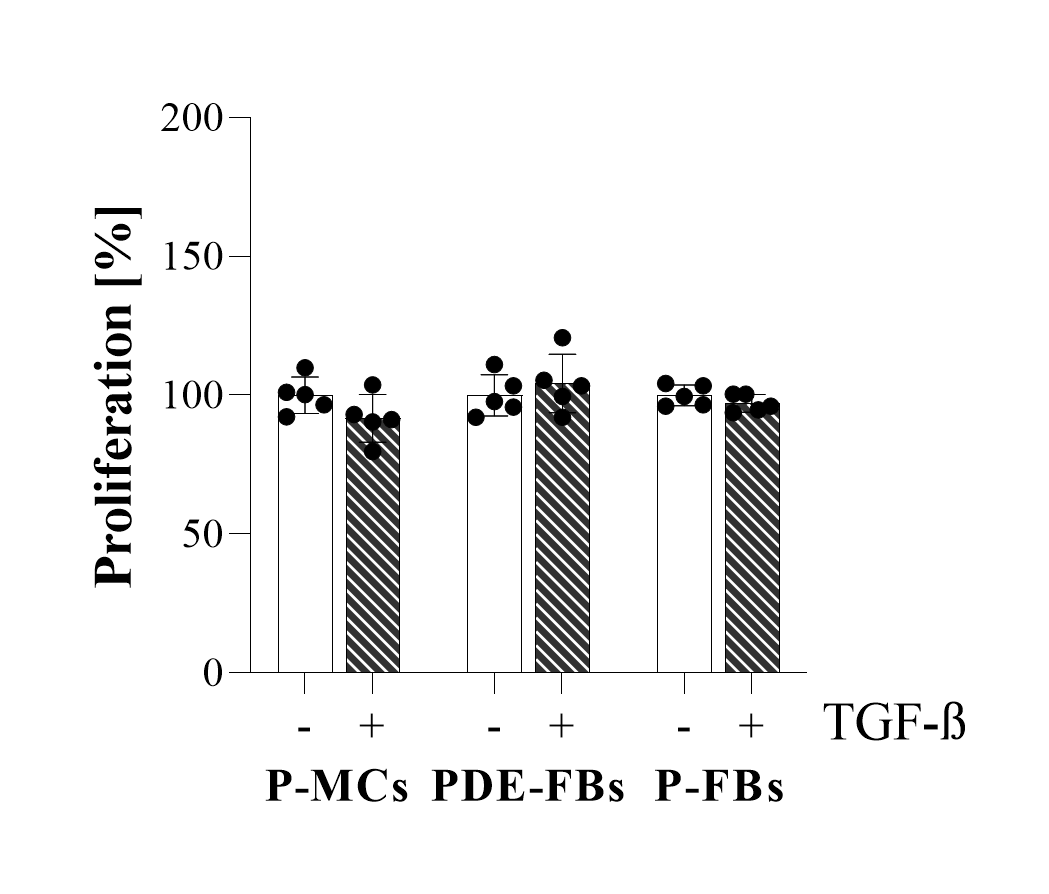

Supplement: Supplementary file 1 [file cells-13-00605-s001.zip › Supl 5.tif]

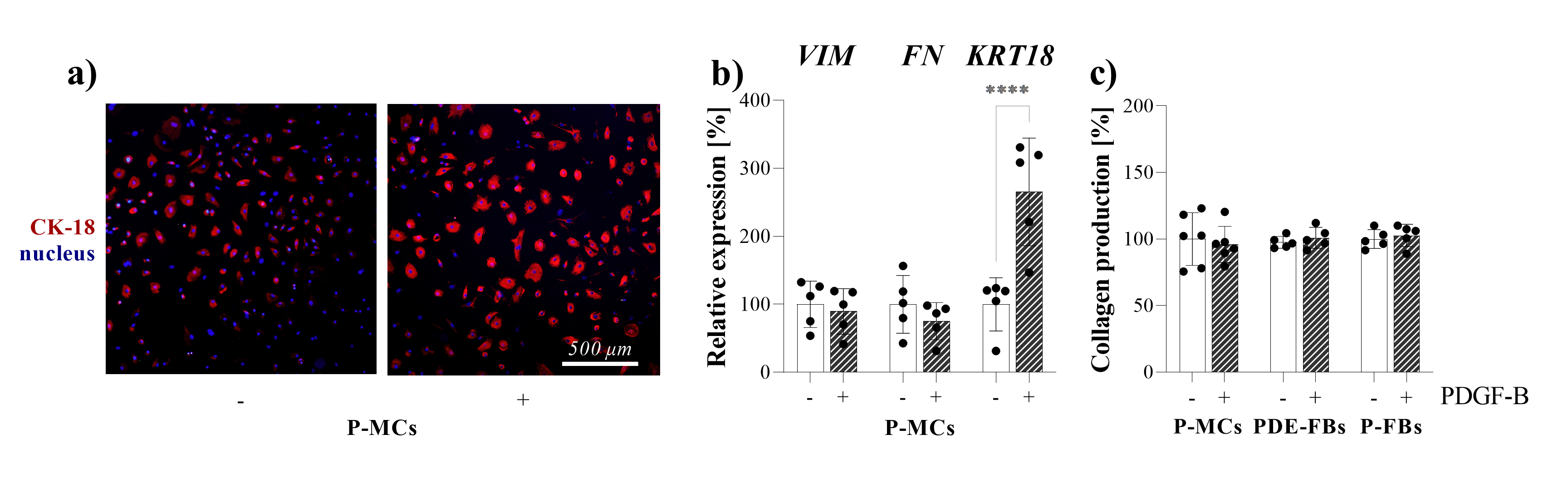

Supplement: Supplementary file 1 [file cells-13-00605-s001.zip › Supl 6.tif]
